# Long-Term Prognosis for Clinical West Nile Virus Infection

**DOI:** 10.3201/eid1008.030879

**Published:** 2004-08

**Authors:** Anne Labowitz Klee, Beth Maldin, Barbara Edwin, Iqbal Poshni, Farzad Mostashari, Annie Fine, Marcelle Layton, Denis Nash

**Affiliations:** * New York City Department of Health, New York City, New York, USA;; † Centers for Disease Control and Prevention, Atlanta, Georgia, USA; 1 Current affiliation: U.S. Department of Veterans Affairs, West Haven, Connecticut, USA; 2 Current affiliation: New York Academy of Medicine, New York, New York, USA

**Keywords:** Activities of Daily Living, Central Nervous System, Encephalitis, Viral, Hospitalization, Human, Meningitis, Viral, Movement Disorders, West Nile Fever, West Nile virus, elderly, research

## Abstract

Relatively little is known about the long-term prognosis for patients with clinical West Nile virus (WNV) infection. We conducted a study to describe the recovery of New York City residents infected during the 1999 WNV encephalitis outbreak. Patients were interviewed by telephone on self-perceived health outcomes 6, 12, and 18 months after WNV illness onset. At 12 months, the prevalence of physical, functional, and cognitive symptoms was significantly higher than that at baseline, including muscle weakness, loss of concentration, confusion, and lightheadedness. Only 37% achieved a full recovery by 1 year. Younger age at infection was the only significant predictor of recovery. Efforts aimed at preventing WNV infection should focus on elderly populations who are at increased risk for neurologic manifestations and more likely to experience long-term sequelae of WNV illness. More studies are needed to document the long-term sequelae of this increasingly common infection.

## Abstract 2

Patients recovering from West Nile virus infection may experience sequelae for months.

West Nile virus (WNV, family *Flaviviridae*, genus *Flavivirus*) has become endemic throughout much of the United States since its introduction in 1999 (1). In 2003, a total of 2,866 laboratory-confirmed human cases of neuroinvasive illness and 264 deaths were caused by WNV infection (2). Older persons are at substantially increased risk for severe WNV disease, a hallmark of which is profound muscle weakness (1), often with acute flaccid paralysis or other motor disorder (2–4).

Investigators of the first WNV disease outbreak in North America in 1999 documented that older persons and persons with diabetes are at increased risk for death after WNV infection (1,5–7) However, few epidemiologic studies have examined the sequelae or time course of recovery from WNV meningitis or encephalitis in survivors. A recent investigation of neurologic manifestations of WNV infections showed persistent symptoms at 8 months after infection, particularly in those patients who experienced flaccid paralysis (8).

We conducted an 18-month follow-up study on a cohort of New York City (NYC) case-patients identified as being ill with WNV infection in 1999 (1). The investigation had the following objectives: 1) to describe the physical, cognitive, and functional outcomes in patients recovering from WNV meningitis or encephalitis over the 18 months after acute illness and 2) to determine whether the severity of the initial clinical syndrome, the patient's age, and the patient's underlying illness affected the likelihood of recovery.

## Methods

The medical records of all patients hospitalized with WNV infections were reviewed by using a standard form to abstract chart information. Follow-up interviews were conducted and blood was collected at approximately 6-month intervals from laboratory confirmed case-patients whose WNV infections were diagnosed in 1999. Three distinct health outcome areas—physical, cognitive, and functional health status—were each assessed at 6, 12, and 18 months after illness onset. Baseline health status was assessed by recall at the 12-month interview. Physical and cognitive health status outcomes were assessed at each interview by asking about the frequency of selected symptoms (Table 1). Functional ability was evaluated by administering the Instrumental Activities of Daily Living Scale (IADLS) (9) to assess daily functioning before and after WNV illness. The prevalence of physical, cognitive, and functional symptoms at baseline (by recall at 12 months) and at the 6-, 12-, and 18-month interviews was calculated. Underlying illness and initial clinical syndrome were ascertained from the medical chart.

**Table 1:** Health outcomes assessed during follow-up telephone interviews of New York residents with clinical West Nile virus infection in 1999^a,b^

Physical health	Cognitive health	Functional health
Difficulty walking^c^ Fatigue Headache Insomnia Joint pain Muscle pain Muscle weakness Seizures Stiff neck	Confusion Depression Irritability Lightheadedness Loss of concentration Loss of memory	Heavy chores Laundry Light housekeeping Managing medications Managing money Meal preparation Shopping Telephoning Transportation

^a^At 12 months post-onset, baseline status for each outcome was assessed; for each outcome, patients were asked to report the degree to which they experienced the signs and symptoms at baseline (by recall) and at 12 months postonset. ^b^Each outcome was scored 0–2 according to the following scale: always = 2, sometimes = 1, never = 0. Functional health was scored according to how frequently the patient had difficulty performing the task. Recovery was calculated as the sum of the baseline score in each category, divided by the sum of the 12-month score. ^c^Difficulty walking was given twice the weight as other outcomes in the recovery score calculation.

The initial clinical syndrome was classified as WNV encephalitis, indicated by fever and altered mental status or other cortical signs (e.g., seizures) and cerebrospinal fluid (CSF) suggestive of viral infection; WNV meningitis, indicated by fever, meningeal signs (documentation of Kernig sign, Brudzinski sign, or nuchal rigidity), and CSF suggestive of viral infection; or WNV fever with headache. CSF suggestive of viral infection was defined as a negative bacterial stain and culture, with elevated leukocyte count (>5 cells/mm^3^) or elevated protein (>4.5 g/L). Proxy interviews were conducted when case-patients could not be interviewed because of poor health, hearing difficulties, or a language barrier.

### Laboratory Methods

Laboratory evidence for recent WNV infection (10) was confirmed in all patients and defined by any of the following test results: 1) isolation of WNV by culture or amplification of WNV RNA by reverse transcriptase–polymerase chain reaction testing from human tissue specimens; 2) demonstration of immunoglobulin (Ig) M antibody to WNV in CSF by IgM-capture enzyme-linked immunosorbent assay (ELISA); 3) greater than fourfold serial change in WNV-specific neutralizing antibody as measured by the plaque-reduction neutralization test (PRNT) in paired, appropriately timed serum samples; or 4) demonstration of both WNV-specific IgM (by ELISA) and IgG (screened by ELISA and confirmed by PRNT) in a single serum specimen. Patients with WNV-specific IgM in a single serum sample were classified as having a probable recent infection. Patients with anti-WNV IgG only in a single serum specimen were also classified as having a probable WNV infection if the antibodies were found to be WNV-specific by PRNT and the patient had no history of travel to an area outside the United States where WNV infection is endemic.

Blood specimens were obtained at 6-month intervals starting at 6 months through 18 months after illness onset, until WNV-specific IgM, indicative of recent infection, was undetectable. Serum samples were tested for anti-WNV IgM (capture ELISA) and IgG (indirect ELISA) (11,12). Results from the ELISA testing were expressed as a WNV-positive to WNV-negative control (P/N) ratio of observed A450 nm (MAC-ELISA) or A405 nm (IgG ELISA) as described. In these tests, P/N ratios >3.0 were considered positive and P/N ratios >2.0 and <3.0 were considered equivocal. Detailed information on WNV serologic features in this cohort study has been previously published elsewhere (13).

### Study Population

Of the 59 surviving patients hospitalized with WNV infection in 1999, 40 were NYC residents and eligible for inclusion in the follow-up study. During the course of the study, two additional patients with laboratory-confirmed WNV infection who had fever and headache were identified and enrolled. Thirty-eight (90.5%) of the 42 case-patients completed the first interview (6 months postonset), 35 (83.3%) participated in the second interview (12 months postonset), and 36 (85.7%) participated in the third interview (18 months postonset). Forty (95%) case-patients participated in at least one of the three interviews; 32 (76%) completed all three interviews. The proportion of interviews that were completed by proxy was 39% at the first interview to 25% at the third interview.

### Recovery Assessment at 12 Months After Infection

A recovery assessment was completed for the 35 case-patients who participated in the 12-month interview. At the 12-month interview, patients responded to questions on the frequency of occurrence (never, sometimes, or often) of selected symptoms during the month before the interview and during the month before illness onset (baseline). A symptom was counted as being present if it was experienced sometimes or often. Current and baseline composite scores were calculated within each health status domain by tabulating the responses for each outcome in that domain. Difficulty walking was weighted double in computing the physical health domain score, because it appears to be a very specific symptom of severe WNV infection (1,3,4). If a response of not applicable or unknown was given for a particular outcome, then that outcome was not included in calculating that case-patient's baseline or 12-month composite score.

Within each health status domain, the ratio of the 12-month composite score to the baseline composite score was calculated and used as a measure of recovery for that domain. Persons with a 12-month composite score >85% of baseline for a given health status domain were considered to be recovered in that domain. Those persons with 12-month composite >85% of baseline in all three health status domains were considered fully recovered.

### Statistical Methods

Prevalence ratios were calculated for all outcomes at each interval relative to baseline; p values associated with prevalence ratios were calculated by using a matched analysis with McNemar test for correlated proportions. Crude and adjusted relative risks (RRs) were calculated to examine the relationships of clinical syndrome (i.e., encephalitis, meningitis, and mild illness), age, and underlying medical conditions with recovery in each health status domain at 12 months postonset. RRs were adjusted by using the method of Mantel and Haenszel. Data were analyzed by using the SPSS System for Windows, version 10.0 and SAS Version 8 (SAS Institute, Cary, NC).

### Consent and Human Subjects Review

Verbal consent was obtained from participants during telephone interviews, and written consent was obtained before each follow-up blood specimen collection. The study protocol underwent human subjects review and was approved by institutional review boards of both the New York City Department of Health and Centers for Disease Control and Prevention.

## Results

Table 2 shows the patients who were ill with WNV infection in 1999 (N = 59) and the 40 surviving NYC residents who were eligible for participation in the follow-up study, plus 2 additional patients with West Nile virus disease who were not hospitalized. Of the 40 surviving NYC case-patients participating in one or more interviews, the median age of the participants at illness onset was 68 years (range: 16 to 90 years), and all patients resided in their own homes before illness. At the time of diagnosis, 22 (55%) patients had encephalitis, 11 (27.5%) had meningitis, and 7 (17.5%) had illness characterized by fever and headache. Of 33 hospitalized patients with known disposition at discharge, those who had diagnoses of encephalitis were more likely to have discharge placements outside their homes (p < 0.05) and more likely to be >65 years of age (p < 0.001).

**Table 2:** Characteristics of participating and nonparticipating patients who survived clinical West Nile virus infection, New York City, 1999

Characteristic	All hospitalized patients, N = 59 (%)	Enrolled patients, N = 42 (%)	Participants in 12-month interview, N = 35 (%)	Nonparticipants in 12-month interview, N = 7 (%)
Age				
<65	23 (39)	16 (38)	13 (37)	3 (43)
>65	36 (61)	26 (62)	22 (63)	4 (57)
Sex				
Female	28 (47)	20 (48)	18 (51)	2 (29)
Male	31 (53)	22 (52)	17 (49)	5 (71)
Underlying illness before infection				
Hypertension	25 (42)	17 (40)	14 (40)	3 (43)
Diabetes	12 (20)	6 (14)	5 (14)	1 (14)
Hypertension or diabetes	31 (53)	19 (45)	16 (46)	3 (43)
Clinical syndrome				
Encephalitis	37 (63)	22 (52)	19 (54)	3 (43)
Meningitis or milder illness	22 (37)	20 (48)	16 (46)	4 (57)
Discharge status^a^				
Dead	7 (12)	NA	NA	NA
Home	22 (37)	20 (50)^b^	20 (61)^c^	NA
Home of family or friend	3 (5)	3 (8)^b^	3 (9)^c^	NA
Skilled nursing facility	4 (7)	4 (10)^b^	4 (12)^c^	NA
Rehabilitation	6 (10)	6 (15)^b^	6 (18)^c^	NA
Unknown but alive	17 (29)	7 (18)^b^	0	7 (100)
Required physical therapy	NA	NA	18 (51)	NA

^a^Includes hospitalized patients only. ^b^N = 40 for these calculations. ^c^N = 33 for these calculations.

### Physical, Cognitive, and Functional Health Status

Table 3 shows the prevalence of physical, cognitive, and functional sequelae reported at 6, 12, and 18 months postonset. At the 12-month interview, patients were also asked to recall the prevalence of those symptoms before illness onset. All participants interviewed with a clinical diagnosis of encephalitis with weakness (N = 10) reported difficulty walking 6 months after illness. Those who had an initial diagnosis of encephalitis were more likely to require a wheelchair at the first follow-up interview than those with meningitis or mild illness.

**Table 3:** Prevalence of signs and symptoms at intervals of follow-up in patients with clinical West Nile virus infection, New York City, 1999

Sign or symptom	Before illness onset^a^ (baseline), n/N (%)	Interview 1 (6 months), n/N (%)	Interview 2 (12 months), n/N (%)	Interview 3 (18 months), n/N (%)	p value for 12 months vs. baseline^b^
Physical sequelae					
Difficulty walking	7/35 (20.0)	30/38 (78.9)	17/35 (48.6)	15/36 (41.6)	0.002
Muscle weakness	4/35 (11.5)	25/38 (65.8)	15/34 (44.1)	20/36 (55.5)	< 0.001
Fatigue	12/35 (34.3)	20/37 (54.1)	22/33 (66.7)	23/36 (63.8)	0.002
Insomnia	7/35 (20.0)	17/38 (44.7)	16/34 (47.1)	17/36 (47.2)	0.007
Muscle pain	12/35 (34.3)	14/37 (37.8)	19/34 (55.9)	14/36 (38.8)	0.035
Headache	9/35 (25.7)	13/37 (35.1)	15/34 (44.1)	13/36 (36.1)	0.014
Joint pain	7/35 (20.0)	12/38 (31.6)	11/34 (32.3)	11/36 (30.6)	0.157
Cognitive symptoms					
Memory loss	7/35 (20.0)	21/38 (55.3)	17/34 (50.0)	16/36 (44.5)	0.002
Loss of concentration	3/35 (8.6)	16/37 (42.2)	14/34 (41.2)	12/36 (33.3)	< 0.001
Depressed	5/35 (14.3)	15/38 (39.5)	13/34 (38.2)	16/36 (44.4)	0.005
Irritable	8/35 (22.9)	14/38 (36.8)	14/34 (41.2)	14/36 (38.9)	0.008
Lightheaded	4/35 (11.5)	13/38 (34.2)	17/33 (51.5)	13/35 (37.1)	< 0.001
Confusion	2/35 (5.7)	17/38 (44.8)	9/34 (26.5)	11/36 (30.6)	0.008
Functional sequelae					
Shopping	4/33 (12.1)	17/36 (47.2)	14/33 (42.4)	14/35 (40.0)	0.002
Meal preparation	2/32 (6.3)	22/31 (71.0)	12/32 (37.5)	12/34 (35.3)	< 0.001
Laundry	1/25 (4.0)	14/29 (48.3)	10/25 (40.0)	10/33 (30.3)	0.003
Light housekeeping	1/28 (3.6)	19/35 (54.3)	12/28 (42.9)	12/35 (34.3)	< 0.001
Heavy chores	5/30 (11.9)	19/33 (57.6)	19/30 (63.3)	19/34 (55.9)	0.003
Transportation	3/29 (10.3)	23/37 (62.2)	10/28 (35.7)	14/36 (38.9)	0.008

^a^Assessed by recall at the 12-month follow-up interview. ^b^Based on McNemar's test for agreement in a matched analysis.

The prevalence of cognitive symptoms was higher 1 year after illness compared with baseline for all cognitive outcomes. All cognitive symptoms were more common after illness onset in case-patients at intervals extending up to 18 months after acute illness (Table 3), and some symptoms did not diminish over time. Prevalence ratios of functional disabilities were also significantly elevated compared with baseline.

### Analysis of Recovery Outcomes

The mean domain-specific health status score was significantly lower at 12 months compared with baseline for all three domains (data not shown). Overall, 54%, 59%, and 57% of patients were physically, cognitively, or functionally recovered, respectively (Table 4). Case-patients >65 years achieved recovery rates of 50%, 52%, and 45% in the respective domains of physical recovery, cognitive recovery, and functional recovery (Table 5). Only 37% of patients were considered fully recovered. Diagnosis (encephalitis versus meningitis or other mild illness) was not predictive of physical or cognitive recovery (Table 4), even after adjusting for age. Age was a positive predictor of recovery in each domain, with younger persons more likely to achieve physical, cognitive, and functional recovery (Table 5). The absence of an underlying health condition was associated with an increased likelihood of recovery in all domains (Table 6). After adjusting for baseline clinical status (Mantel-Haenszel method), younger persons (<65 years) were significantly more likely to achieve a full recovery than older persons (>65 years) (relative risk [RR] = 3.3, 95% confidence interval [CI] 1.1–9.9). After adjusting for underlying illness, younger persons were also more likely to recover fully than older persons (RR = 2.3, 95% CI 0.97–5.5).

**Table 4:** Recovery at 12 months post-onset by health status domain and clinical syndrome at diagnosis in patients with clinical West Nile virus infection, New York City, 1999

Recovery	Total	Recovered, n (%)^a^	Not recovered, n (%)^a^	Risk ratio	95% confidence interval
Physical recovery					
Meningitis or mild illness	16	8 (50.0)	8 (50.0)	0.86	0.46–1.6
Encephalitis	19	11 (57.9)	8 (42.1)	Referent	
Total	35	19 (54.3)	16 (45.7)		
Cognitive recovery					
Meningitis or mild illness	16	10 (62.5)	6 (37.5)	1.1	0.64–2.0
Encephalitis	18	10 (55.5)	8 (44.4)	Referent	
Total	34	20 (58.8)	14 (41.2)		
Functional recovery					
Meningitis or mild illness	16	10 (62.6)	6 (37.5)	1.2	0.67–2.1
Encephalitis	19	10 (52.6)	9 (47.4)	Referent	
Total	35	20 (57.1)	15 (42.9)		
Total recovery					
Meningitis or mild illness	16	7 (43.8)	9 (56.3)	1.4	0.58–3.3
Encephalitis	19	6 (31.6)	13 (68.4)	Referent	
Total	35	13 (37.1)	22 (62.9)		

^a^Due to rounding, not all values add up to 100%.

**Table 5:** Recovery at 12 months post-onset by health status domain and age at illness onset in patients with clinical West Nile virus infection, New York City, 1999

Recovery	Total	Recovered, n (%)^a^	Not recovered, n (%)^a^	Risk ratio	95% confidence interval
Physical recovery					
<65	13	8 (61.5)	5 (38.5)	1.2	0.68–2.2
>65	22	11 (50.0)	11 (50.0)	Referent	
Total	35	19 (54.3)	16 (45.7)		
Cognitive recovery					
<65	13	9 (69.2)	4 (30.8)	1.3	0.77–2.3
>65	21	11 (52.4)	10 (47.6)	Referent	
Total	34	20 (58.8)	14 (41.2)		
Functional recovery					
<65	13	10 (76.9)	3 (23.1)	1.7	0.98–2.9
>65	22	10 (45.5)	12 (54.5)	Referent	
Total	35	20 (57.1)	15 (42.9)		
Total recovery					
<65	13	8 (61.5)	5 (38.5)	2.7	1.1–6.5
>65	22	5 (22.7)	17 (77.3)	Referent	
Total	35	13 (37.1)	22 (62.9)		

^a^Due to rounding, not all values add up to 100%.

**Table 6:** Recovery at 12 months postonset by health status domain and underlying health condition in patients with clinical West Nile virus infection, New York City, 1999

Recovery	Total	Recovered, n (%)^a^	Not recovered, n (%)^a^	Risk ratio	95% confidence interval
Physical recovery					
No underlying condition	18	11 (61.1)	7 (38.9)	1.3	0.70–2.4
Hypertension or diabetes	17	8 (47.1)	9 (52.9)	Referent	
Total	35	19 (54.3)	16 (45.7)		
Cognitive recovery					
No underlying condition	17	11 (64.7)	6 (35.3)	1.2	0.70–2.2
Hypertension or diabetes	17	9 (52.9)	8 (47.1)	Referent	
Total	34	20 (58.8)	14 (41.2)		
Functional recovery					
No underlying condition	18	12 (66.7)	6 (33.3)	1.4	0.78–2.6
Hypertension or diabetes	17	8 (47.1)	9 (52.9)	Referent	
Total	35	20 (57.1)	15 (42.9)		
Total recovery					
No underlying condition	18	9 (50.0)	9 (50.0)	2.1	0.80–5.6
Hypertension or diabetes	17	4 (23.5)	13 (76.5)	Referent	
Total	35	13 (37.1)	22 (62.9)		

^a^Due to rounding, not all values add up to 100%.

## Discussion

We report that WNV infection can result in a protracted convalescent period with long-term physical, cognitive, and functional impairments lasting >18 months after acute illness. Approximately 40% of patients hospitalized in 1999 did not return to their own homes immediately after discharge, and physical therapy was required by 47% of patients after hospitalization. Comparing the prevalence of symptoms before illness with that at 12 months after WNV illness onset, physical, functional, and cognitive symptoms persisted. We estimate that 37% achieved full recovery by 12 months. Younger age (<65 years) was the only significant predictor of achieving a full recovery.

WNV is clinically, serologically, and epidemiologically similar to St. Louis encephalitis virus (SLEV) (14–18), and recovery after WNV infection might be comparable to that of patients recovering from SLEV-associated encephalitis (SLE). Information on sequelae from SLE has been documented after U.S. outbreaks occurring from the 1930s to the 1970s. Various methods assessed recovery from SLE, including medical examinations (with neurologic assessments) and patient or proxy interviews (19). Follow-up times varied from 6 months to 5 years after acute illness (15–23). In general, studies of recovering patients with SLE have documented generalized susceptibility to fatigue, headaches, nervousness, inability to concentrate, depression, and problems with gait and balance throughout convalescent periods of 6 months to 3 years after acute SLEV infection; on average, ≈30% of case-patients were not fully recovered 1 year after acute illness (19,20,24).

Different approaches to defining recovery were used by researchers who characterized the experience of patients after SLEV infection. After the first SLE epidemic in St. Louis in 1933, researchers defined overall recovery based on the ability to return to work. Of 331 patients, 141 (66%) reported that they felt completely recovered 12–18 months after acute illness, whereas 22 (6.7%) felt they were physically unable to return to their jobs. Although none of the patients <20 years of age was incapacitated, >10% of patients >20 years could not return to work (20). After an SLE epidemic in Mississippi in 1975, researchers conducted follow-up interviews 6 months after illness onset. Of the 175 patients contacted, 87 (49.7 %) achieved full recovery, 24 (13.7%) reported minor symptoms, and 29 (16.6 %) reported that they resumed previous activities but not at the same level. SLE patients from the Tampa Bay, Florida, outbreaks occurring from 1959 to 1962 (N = 160) had more difficulty completing tests that evaluated balance and equilibrium than controls. In particular, SLE patients had difficulty walking in straight lines and widening their lateral base of support (25). Predominant cognitive problems included nervousness, irritability, depression, and forgetfulness (15–23).

Our findings are similar to those reported in these SLE studies. Regardless of acute clinical symptoms, WNV case-patients in this study continued to report difficulty walking, muscle weakness, fatigue, and insomnia, with >40% reporting a combination of these difficulties, and 30% continued to report persistence of memory loss, confusion, depression, and irritability at 18 months after acute illness. Eighteen months after illness, 30% of case-patients reported needing assistance with activities of daily living, mostly those requiring increased strength. Although average functional ability from 6 months to 1 year post-onset improved significantly, functional ability reached a plateau and did not improve further during the 12- to 18-month period.

Our results suggest that WNV has more severe long-term sequelae in older persons than in younger persons. These sequelae may be attributable to the severity of the patients' WNV infection, to the more general effects of serious illness and hospitalization, or to the aging process itself; regardless, WNV causes severe neurologic illness and might be associated with lasting sequelae in persons >65 years.

The presence of underlying disease at the time of onset of illness was not significantly associated with recovery at 12 months (RR = 1.4, 95% CI 0.58–3.3), even after adjusting for age (adjusted RR = 1.3, 95% CI 0.70–2.5). However, the lack of significance of this association could be a result of the small number of patients in our study or misclassification.

Several aspects of our investigation might limit the generalizability of these findings. Although participation was high, our estimates may be imprecise because of the small sample size. Furthermore, the ages of the study participants span a wide range (16–90 years), making adequate adjusting for age difficult. We used a structured interview questionnaire, the content and format of which, when possible, was similar across interviews to maximize comparability of data obtained over time. Proxies were used when case-patients could not be interviewed because of poor health, hearing difficulties, or a language barrier. Data were based on subjective report, either by the patient or their proxy. Subjective accounts provided by persons who are cognitively impaired might overattribute or underattribute certain dysfunctions to their WNV illness, and recall bias might have caused case-patients to selectively suppress or exaggerate information about their health status, either current or past.

Baseline information regarding physical, cognitive, and functional health before WNV disease was collected during the second follow-up interview at 1 year (i.e., by recall). Participants may have had problems recalling baseline health status over a 12-month period, limiting our ability to accurately ascertain actual baseline level of functioning. Sequelae could not be verified by objective physical examination, physician interview, or medical record review. Future studies of recovery in WNV patients should attempt to obtain more objective measurements of sequelae, such as provider interviews, medical chart review, or neurologic examination. As WNV continues to affect older age groups, further research should consider ways to control for declines in functioning associated with the aging process and to obtain objective data regarding baseline status. Finally, future studies should try to assess the baseline health status of WNV patients closer to the time of onset to reduce the impact of recall bias on long-term measures of recovery.

Our study documents that, in addition to causing severe acute illness, WNV meningitis or encephalitis likely results in a prolonged recuperation and rehabilitation period, especially in older persons. As WNV continues to establish itself as a national public health concern, these findings reinforce the need for local governments in affected areas to institute widespread public health measures to safeguard against WNV transmission and for persons—especially those age 65 and over—to take precautions to avoid exposure to mosquitoes and reduce mosquito breeding sites on their properties. More studies are needed to document the long-term sequelae of this increasingly common infection.
